# Coronary Artery Perforation and Regrowth of a Side Branch Occluded by a Polytetrafluoroethylene-Covered Stent Implantation

**DOI:** 10.5402/2011/212851

**Published:** 2011-04-26

**Authors:** Shams Y-Hassan, Christer Sylvén, Loghman Henareh

**Affiliations:** Department of Cardiology, Karolinska Institute at Karolinska University Hospital, Huddinge, 141 86 Stockholm, Sweden

## Abstract

Stenting of the right coronary artery stenosis caused coronary perforation and profound dye (blood) extravasation in a 69-year-old female patient. Instantaneous balloon inflation followed by implantation of a polytetrafluoroethylene- (PTFE-)covered stent sealed the coronary perforation, restored the blood flow, and perceivably caused acute occlusion of a large side branch (SB). The immediate in situ balloon inflation prevented the development of cardiac tamponade. Surprisingly, followup coronary angiography 4 and 11 months later showed spontaneous recanalization of the SB occluded by PTFE-covered stent. The SB was filled through a channel beginning at the end of the covered stent streaming retrogradely beneath it toward the SB ostium. Up to the best of our knowledge, this is the first described case of late spontaneous recanalization of as SB occluded by a PTFE-covered stent.

## 1. Introduction

Coronary artery perforation is a rare but life-threatening complication of percutaneous coronary intervention (PCI) [1, 2]. Among the measures used to treat coronary perforation is the implantation of polytetrafluoroethylene- (PTFE-) covered stent, which will seal the perforation and perceivably occlude the side branches (SBs) originating from the stented region [3]. Spontaneous recanalization of an occluded SB is common after implantation of both bare-metal and drug-eluting stents, but it has not been reported after occlusion by PTFE-covered stent implantation [4]. Herein and to the best of our knowledge, for the first time, we report on a patient with spontaneous late recanalization of a SB occluded by a PTFE-covered stent implanted to seal a coronary perforation.

## 2. Case Presentation

**Figure 1:**
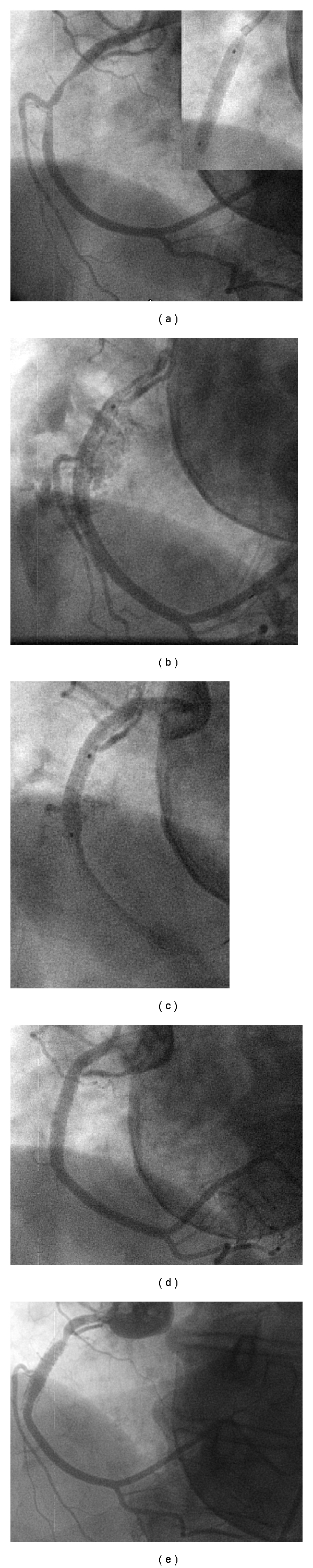
(a) Left anterior oblique projection demonstrating right coronary artery (RCA) stenosis and stent implantation. Right ventricular branch (side branch (SB)) emanates from the stenosed region. (b) contrast leakage through a relatively large hole into the pericardium after stent deployment. (c) contrast leakage is reduced markedly after immediate and prolonged balloon inflation. (d) complete cessation of contrast extravasation after polytetrafluoroethylene- (PTFE-) covered stent implantation with occlusion of the SB. (e) followup angiography of the RCA 4 months later reveals moderate instent restenosis and regrowth of the SB occluded by the PTFE-covered stent.

**Figure 2:**
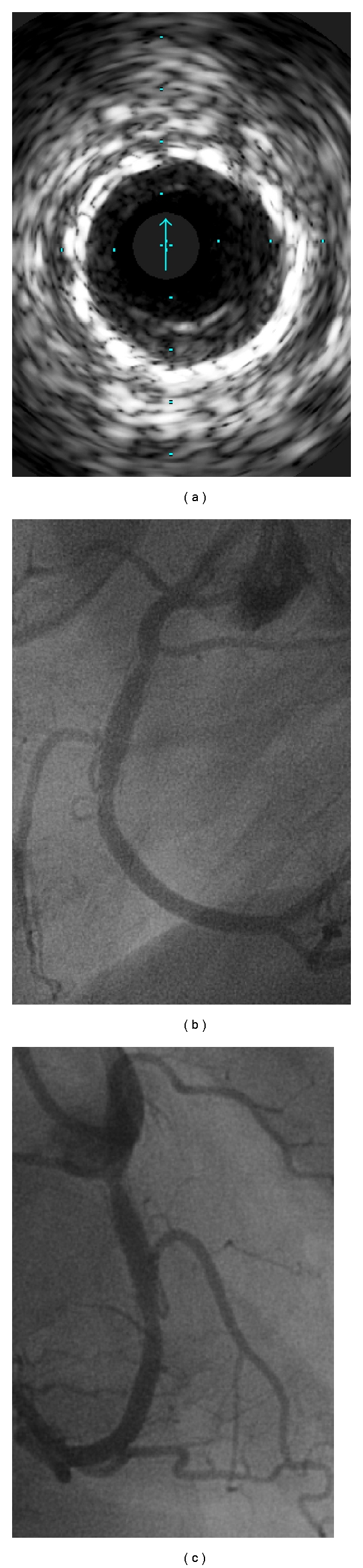
Intravascular ultrasound (IVUS) (a) shows moderate hyperplasia of the intima and well-apposition of the PTFE-covered stent to the first implanted stent. Right coronary angiography in caudal left anterior oblique (b) and in right anterior oblique projection (c) shows the channel between the right coronary artery and the side branch. Both IVUS and right coronary angiography were done 11 months after the incident.

The case is a 69-year-old female patient with angina pectoris. Her previous history was unremarkable apart from bilateral cosmetic implantation of breast prosthesis many years ago. Coronary angiography revealed mild changes in the left coronary artery and a significant stenosis in the right coronary artery (RCA). A relatively large SB, which was the right ventricular branch, emanated from the stenosed region of the RCA. The stenosis was dilated with an undersized balloon followed by implantation of a bare-metal stent-type driver 4.0 × 24 mm with a pressure of 14 atmospheres (Figure 1(a)). This resulted in acute right coronary perforation and profound dye (blood) extravasation (Figure 1(b)). The stent balloon was inflated instantaneously at the perforation site with a relatively low pressure for a period of about 2 minutes whilst preparing a PTFE-covered stent. The immediate and prolonged balloon inflation reduced the dye extravasation markedly (Figure 1(c)) and prevented development of cardiac tamponade. The largest available PTFE-covered stent 3.5 × 19 mm was successfully implanted. The covered stent was then postdilated with a 4 mm noncompliant balloon with a pressure up to 22 atmospheres. This successfully sealed the perforation with complete cessation of dye extravasation and restoration of normal anterograde flow (Figure 1(d)). The graft stent intelligibly resulted in the SB occlusion. Repeated echocardiography revealed moderate pericardial effusion but no signs of early or late cardiac tamponade. Followup angiography of the RCA four months (Figure 1(e)) and, because of increasing dyspnea, 11 months after the incident showed a moderate instent restenosis at the proximal and distal edges of the covered stent. The fractional flow reserve measurement under adenosine infusion was 0.93 distal to the moderate instent restenoses. To our surprise, the SB was recanalized after occlusion by the PTFE-covered stent (Figure 1(e)). Intravascular ultrasound (IVUS) examination showed that the covered stent was well apposed to the first stent. The SB and its ostium could not be seen by IVUS (Figure 2(a)). Critical and frame-after-frame review of the angiography in different projection showed that the SB is filled through a channel beneath the PTFE-covered stent. The channel begins at the distal end of the covered stent and continues upward retrogradely toward the SB ostium filling the SB (Figures 2(b) and 2(c)). 

## 3. Discussion

Coronary artery perforation is a rare but life-threatening complication of PCI. It may lead to significant morbidity and mortality including cardiac tamponade and death [1]. Ellis group III coronary perforation (perforation ≥ 1 mm in diameter with contrast streaming) is associated with higher rates of myocardial infarction, cardiac tamponade, and need for emergency coronary artery bypass graft surgery [2]. The most common causes of coronary perforation are guide wire (especially hydrophilic) manipulation, coronary stenting, angioplasty alone, and the use of atheroablasive devices [1]. The treatment is to seal the perforation with prolonged balloon inflation, covered stent implantation (sometimes noncovered), pericardiocentesis, and emergency cardiac surgery [3]. The PTFE-covered stent will seal the perforation and understandably occlude the side branches originating from the stented region.

The stent balloon was still in place in our case when the coronary perforation was detected. The balloon was inflated immediately while preparing the covered stent. The instantaneous balloon inflation prevented the development of cardiac tamponade. This highlights the importance of having the stent balloon in place while checking the immediate results of stent implantation at the lesion site. 

Side branch occlusions have been reported during implantation of both bare-metal (7%) and drug-eluting (10%) stents. Spontaneous recanalization of an occluded SB is common after implantation of both bare-metal (67%) and drug-eluting (92%) stents [4]. Recanalization of an occluded SB after a PTFE-covered stent implantation has not been reported previously. Our patient is the first described case to show regrowth of an occluded SB after a PTFE-covered stent implantation. Critical review of the angiographic pictures taken 4 months after the index procedure showed that the SB is not filled directly from the RCA lumen at the level of its origin but shortly after the contrast has passed the ostial level of the SB. The possibility that the contrast penetrates a blood impermeable membrane like PTFE was unlikely. It is not possible for PTFE compounds to break down at human temperatures. There were no signs of collateral flow from another branch to the SB. Intravascular ultrasound (IVUS) could not show any sign of flow to the SB at the level of its ostium, and PTFE-covered stent was well apposed to the first implanted stent (Figure 2(a)). Self-critical analysis of the possible events that could have led to recanalization of the SB is blood flow around the covered stent toward the SB or stent dislocation. The reason for consideration of these two possibilities is that the covered stent used in this case was 3.5 mm in diameter (larger than 3.5 mm in diameter was not available in our lab) which is a half mm smaller than the first stent that caused coronary perforation. The covered stent was postdilated with a 4 mm noncompliant balloon at a high pressure to a diameter of more than 4 mm (up to 4.13 mm at pressure of 20 atmospheres). In spite of postdilatation, blood flow through a channel between the 2 stents filling the SB cannot be excluded and may be the most plausible explanation for recanalization of the SB. Graft stent dislocation could be excluded because of the fixed unchanged spatial relation of the graft stent to some angiographic land markers as the first stent and the origin of the SB. Right coronary angiography done 11 months after the index procedure confirmed our first possibility, where frame-after-frame analysis of the angiography in different projections showed that the SB fills through a channel around the PTFE-covered stent. The channel begins at the distal end of the covered stent and courses backward under the covered stent toward the ostium of the SB.

In conclusion, we have described for the first time a case of spontaneous recanalization of a SB occluded by a blood impermeable PTFE-covered stent. We also would like to emphasize the importance of checking the coronary artery immediately after stent implantation while the stent balloon is in place. This helped us in our case to prevent development of cardiac tamponade.

##  Conflicts of Interests 

We would like to confirm that we have no conflict of interests regarding the paper.
